# Reduced Preparatory Responses in People with Dementia Predict Caregiver Mental Health Improvements After Care Ends

**DOI:** 10.1093/geroni/igaf122.1644

**Published:** 2025-12-31

**Authors:** Julian Scheffer, Kuan-Hua Chen, Claire Yee, Jenna Wells, Sandy Lwi, Enna Chen, Jennifer Merrilees, Robert Levenson

**Affiliations:** University of Western Ontario, London, Ontario, Canada; University of Nebraska Medical Center, Omaha, Nebraska, United States; Mayo Clinic, Scottsdale, Arizona, United States; Cornell University, Ithaca, New York, United States; VA Northern California Health Care System, Fairfield, California, United States; Stanford University, Stanford, California, United States; University of California, San Francisco, San Francisco, California, United States; University of California, Berkeley, Berkeley, California, United States

## Abstract

Caregivers for people with neurodegenerative disease (PWNDs) often experience mental and physical health problems, especially when caring for PWNDs who have deficits in emotional functioning. We studied 65 PWNDs and their caregivers both during active caregiving and after caregiving had ended to determine: (a) how caregiver health changes after caregiving ends; and (b) whether PWNDs’ emotional functioning predicts these changes. PWND emotional functioning was assessed in the laboratory by measuring their generation of preparatory physiological activity (e.g., increased heart rate) to an upcoming emotional event (i.e., emotion eliciting films). Caregiver mental and physical health were measured using the Short-Form Health Survey (SF36) during active caregiving and again after caregiving had ended. Results indicated that, overall, caregivers reported increases in mental health, (t(36) = 2.58, p = .014, 95% CI [1.31, 10.93]) but decreases in physical health (t(36) = -2.35, p = .025, 95% CI [-7.26, -0.53]) across the two measurement periods. Using latent change score models, PWNDs’ preparatory physiological response impairments were associated with better caregiver mental health trajectories (β = -0.37, SE = 0.04, p = .003) such that smaller PWND preparatory responses predicted greater improvements in caregiver mental health (β = 9.92, SE = 2.84, p < .001). There was no association with changes in caregiver physical health. Smaller preparatory physiological responses in PWNDs may indicate diminished emotional responding, which is linked with lower caregiver mental health during active caregiving. Once caregiving ends, these caregivers may be most likely to “rebound” and show improvements in mental health.

